# Investigating the effects of construction industry noise on workers’ cognitive performance and learning efficiency

**DOI:** 10.3389/fnhum.2025.1549824

**Published:** 2025-03-17

**Authors:** Xinying Cao, Yian Lu, Decheng Zheng, Peicheng Qin

**Affiliations:** School of Civil Engineering and Architecture, Hainan University, Haikou, China

**Keywords:** construction workers, noise, learning efficiency, cognitive state, electroencephalogram

## Abstract

Despite growing industrialization, the cognitive and psychological impacts of construction noise on workers remain inadequately addressed in empirical research. This study examines the impact of different noise types and intensities on the cognitive performance and learning efficiency of construction workers, using electroencephalogram (EEG) and behavioral data. Specifically, it analyzes the effects of complex noise and steady noise on workers’ attention, mental workload, mental fatigue, and mental stress. The results indicate that complex noise significantly reduces learning efficiency, notably impairing accuracy and reaction time relative to steady noise. This adverse effect is attributed to the unpredictability and variability of complex noise, which disrupts workers’ cognitive processing and heightens mental fatigue. In contrast, although steady noise does not significantly impact mental workload, it induces greater mental fatigue and mental stress than complex noise, especially at high noise levels. The findings also reveal that workers develop some level of adaptation to continuous noise, mitigating its overall impact on learning efficiency. However, elevated noise levels, regardless of type, consistently lead to significant declines in attention and increases in mental stress and mental fatigue. This research makes an original contribution by providing evidence-based insights into the interaction between noise characteristics and worker cognition, offering practical implications for targeted noise management strategies to improve learning efficiency and well-being in construction environments.

## 1 Introduction

The development of the construction industry is fundamentally dependent on its extensive workforce. However, due to limited skill levels and insufficient knowledge, many construction workers often struggle to adapt to the demands of industrialized construction, particularly the operation of intelligent equipment and the requirements of precision production. This skills gap poses a significant barrier to the transition from traditional construction roles to those suited for industrialized construction processes, becoming a major obstacle to the industry’s transformation and upgrading. Therefore, enhancing the knowledge and skill levels of construction workers has become one of the critical challenges that must be addressed to facilitate the industry’s transformation and advancement. Despite various re-education and skill training programs implemented by the government and enterprises, workers’ participation remains low, and the effectiveness of these initiatives is limited (Sun and Wang, 2017).

In practice, construction workers primarily acquire new skills through a “learning while working” approach, which directly ties their learning efficiency to the work environment. Discrepancies between controlled training environments and the more complex, real-world conditions often hinder workers’ ability to adapt quickly and effectively, necessitating additional learning and adjustment periods (Xiong et al., 2018). Thus, it is essential to deeply analyze the factors influencing training effectiveness within the actual work environments of construction workers to better support their transition to industrialized roles.

Noise is one of the most hazardous factors in construction work environments. Beyond its direct physiological harm, noise significantly affects workers’ psychological states and overall work efficiency, thereby reducing productivity (Lie et al., 2016). Prolonged exposure to high-decibel noise environments can lead to temporary or permanent hearing loss, while also contributing to cardiovascular issues such as hypertension and arrhythmias (Skogstad et al., 2016). Additionally, long-term exposure to noise can induce anxiety, tension, and stress anxiety, tension, and stress, which diminish job satisfaction and further negatively impacts their job performance and productivity (Basner et al., 2014). Noise also disrupts concentration, resulting in lapses in attention that compromise decision-making, reduce work efficiency, and elevate the risk of workplace accidents (Singh et al., 2010).

In the construction industry, noise has a pervasive and profound impact on the learning efficiency of construction workers. Continuous exposure to noise disrupts essential cognitive abilities such as attention and memory, imposing additional mental workload when workers attempt to learn new skills or comprehend complex information. This results in even simple training content requiring longer time to master (Lan, 2010; Xiong et al., 2018). Furthermore, noise-induced hearing impairments and communication barriers hinder effective knowledge transfer, reducing team collaboration efficiency. Chronic noise exposure also adversely affects sleep quality, thereby impairing daytime alertness and learning capacity. Over time, persistent psychological stress and anxiety induced by noise diminish workers’ motivation and focus, leading to lower job satisfaction and reduced participation in training activities (Smith, 1990; Singh et al., 2010).

Research on the impact of noise in the construction industry has traditionally focused on hearing loss, protective measures, and noise control management. Most of these studies rely on subjective survey methods, such as questionnaires and interviews, which are more susceptible to subjective biases. Previous research has demonstrated that individuals’ subjective perceptions of noise can vary over time, with individuals alternating between categorizing the same sounds as noise or non-noise depending on the period (Fernandez et al., 2009). Given the limitations of subjective approaches, physiological measurement tools offer a more objective assessment of noise’s impact on cognitive states. Common physiological measurement tools include electroencephalogram (EEG), heart rate variability (HRV), eye tracking, and skin conductance.

In recent years, the rapid development and increasing availability of portable EEG devices and data processing algorithms have produced significant findings on the impact of noise in the construction industry. For instance, Chen et al. (2016) used EEG to calculate the cognitive states of construction workers and assess the risks they face while performing tasks, thereby helping to reduce accident rates. Qi et al. (2021) evaluated the interference of industrial noise on workers’ cognitive states by analyzing power spectra from various frequency bands during noise exposure. Ke et al. (2021) explored how different noise conditions affected workers’ behavioral performance in safety recognition tasks, examining cognitive state indicators such as attention and mental workload using EEG metrics. Similarly, Mir et al. (2022) studied the impact of different levels and types of construction noise on emotional states using EEG, aiming to reduce the adverse impacts of noise on its recipients.

Despite these advancements, most EEG-based research has primarily concentrated on construction safety, with limited focus on understanding how construction noise affects workers’ learning and training. This oversight represents a critical gap in the literature, as cognitive performance in learning environments is crucial for the development and skill acquisition of construction workers. In the context of noise research within the construction industry, the use of EEG signal features to evaluate the impact of noise on cognitive states has garnered increasing attention from scholars. Nevertheless, the majority of studies remain centered on safety-related issues, while investigations into the effects of noise on learning efficiency and training processes have been relatively sparse.

Therefore, this study employs non-invasive portable EEG devices to collect brainwave data, aiming to address the impact of noise on learning and training during the transition of construction workers to industrialized roles. The aim is to evaluate the effects of various noise levels and types on the learning efficiency and cognitive states of construction workers. By deepening the understanding of how industrial noise affects cognitive functions, this research aims to mitigate its adverse impacts and improve the overall effectiveness of learning and training programs for workers. Ultimately, the findings will provide critical insights to inform decision-making processes to expedite the transition of construction workers to industrialized construction roles.

## 2 Literature review

### 2.1 Research on learning and training of construction workers

With the continuous advancement of the construction industry toward industrialization, facilitating the transformation of construction workers into industrialized construction workers has emerged as a pivotal trend in the development of the industry (Wei et al., 2012). This shift requires not only technological and managerial innovations but also places a significant emphasis on enhancing workers’ skills and knowledge through learning and training initiatives.

On the one hand, numerous scholars are vigorously exploring and applying cutting-edge technologies with the aim of revolutionizing traditional training models. Advanced technologies such as artificial intelligence (AI), virtual reality (VR), and augmented reality (AR) are driving significant improvements in conventional training methods. Tang et al. (2023) found the traditional worker training overly theoretical with subpar results. They thus introduced a “Building Information Modeling (BIM) + VR” immersive setup, enhancing training autonomy and learning effectiveness. Similarly, multiple studies have furthered VR-related training advancements. Frédéric et al. (2015) combined mixed reality (MR), VR, and visualization for better learning efficiency and willingness. Osti et al. (2021) created a VR-based simulated environment that outperformed 2D video training in knowledge retention, task performance, learning speed, and engagement. Bao et al. (2022) proposed an IFC-and immersive network-based cross-platform framework. This allowed workers to engage in VR safety training via mobile or desktop, with real-time interaction to boost efficiency. Collectively, these efforts demonstrate the significant potential of VR-integrated technologies in enhancing construction worker training. On the other hand, certain scholars are integrating new technologies into traditional training and research models. For example, Zhou et al. (2021) integrated VR into traditional safety training, enhancing construction workers’ cognitive abilities and reducing unsafe behaviors. Shi et al. (2019) used VR to study behavioral demonstrations in hazardous sites, finding positive ones promoted safety while negative ones led to more errors. Lin et al. (2018) applied Three-Dimensional (3D) visualization to break communication barriers for low-literacy workers, improving training efficiency. Hasanzadeh et al. (2017) utilized eye-tracking in safety training, boosting both training and workplace efficiency. These studies showcase various ways of using technologies to enhance construction worker training and safety.

Moreover, some researchers focused on enhancing workers’ learning and training effectiveness by improving educational content and optimizing courses. Xu et al. (2019) emphasized individual differences among construction workers, suggesting personalized safety training for better efficiency and performance. Chen et al. (2020b) identified subjective norms as key in construction workers’ learning, proposing norm-based management in training programs. Pham et al. (2023), using the Theory of Planned Behavior, found different influencing factors on learning intentions for non-managerial or non-professional workers. Duan et al. (2024) proposed a neural-network-based character approach to generate safety labels, recommend materials, and boost worker participation and safety training effectiveness.

### 2.2 The impact effects of noise in construction workplaces

Traditional noise assessment typically relies on sound pressure level as a key indicator. Sound pressure refers to the force exerted by sound waves as they vibrate through the air, reflecting the intensity of the sound. It is measured in decibels [dB(A)]. Research has shown that when noise levels exceed 70 dB(A), significant physiological changes occur in subjects. Noise levels between 35 dB(A) and 65 dB(A) can cause annoyance, while noise levels ranging from 66 dB(A) to 85 dB(A) can trigger bodily alarm responses, potentially leading to hearing loss (Seidler et al., 2017; Golmohammadi et al., 2022). Noise in the workplace can negatively impact health, including both auditory and non-auditory effects.

The most common auditory effect of noise is Noise-Induced Hearing Loss (NIHL), which, in severe cases, can result in hearing disability (Li et al., 2016; Le et al., 2017). High noise levels continuously stimulating the cochlea damage ear hair cells, which cannot regenerate, leading to permanent hearing loss under prolonged exposure. Multiple studies across various industries, such as a manufacturing enterprise study by Rn et al. (2022) showing increased detection rates of hearing loss and hypertension with age and noise exposure duration, a petrochemical companies’ risk assessment by Deng et al. (2022) revealing permanent threshold shifts in hearing for noise-affected workers, and those in the automotive manufacturing industry (Huang et al., 2013; Bao et al., 2019; Chen et al., 2020c) indicating that longer exposure and higher intensities increase the likelihood of hearing loss, along with Lie et al.'s (2016) systematic review finding an occupational noise-induced hearing loss rate of 7–21% with higher risks in sectors like manufacturing, shipbuilding, construction, military, and agriculture, all confirm the significant impact of noise on hearing. Additionally, Chen et al. (2020a) summarized the epidemiology, pathogenesis, and preventive measures for occupational noise-induced hearing loss, stressing the importance of noise prevention programs like reducing noise production or guiding proper use of Hearing Protection Devices (HPDs). Non-auditory effects of noise refer to health impacts beyond damage to the auditory system, encompassing a wide range of conditions such as noise-induced annoyance, cardiovascular diseases, impaired cognitive performance, sleep disturbances, and changes in neurobehavioral functions (Luo et al., 2005). In a study by Skogstad et al. (2016) investigating the incidence and mortality of cardiovascular diseases linked to occupational noise exposure, it was found that long-term exposure to workplace noise increases the risk of cardiovascular diseases. Workers exposed to noise levels above 85 dB(A) showed statistically significant risk estimates for hypertension, cardiovascular diseases, and cardiovascular mortality. Noise-induced annoyance typically arises from noise interfering with daily activities, rest, or sleep, and the accompanying negative emotions increase the risk of disease (Basner et al., 2014). Prolonged and high-intensity occupational noise exposure can lead to neurobehavioral changes, such as reduced sensitivity to visual and auditory stimuli, increased reaction time, and deteriorated cognitive abilities, particularly memory (Gomes et al., 1999). Numerous studies have demonstrated that cognitive performance is significantly affected in noisy environments, increasing mental stress and altering mental workload (Jung et al., 2009; Irgens-Hansen et al., 2015; Hao and Conway, 2022).

While construction industry research has conventionally centered on hearing loss, protective measures, and noise control management (Arezes and Miguel, 2005; Lewkowski et al., 2018), it’s crucial to note that noise affects speech comprehension, shortens attention span, decreases work and learning efficiency, heightens work pressure, and raises error rates, thus reducing productivity and increasing production costs (Sörqvist, 2014; Ruan et al., 2016; Vassie and Richardson, 2017); in the manufacturing industry, noise is classified as steady or complex, with complex noise being steady noise overlaid by high-energy transient impulse noise, and epidemiological studies and animal experiments showing it often causes more hearing loss than steady noise due to its irregular temporal patterns and sudden high-energy bursts (Stephenson, 2017), and construction site noise perception surveys indicating that construction equipment with complex noise characteristics like drills and crushers causes more annoyance to workers (Lee et al., 2019).

### 2.3 EEG-based measurement of cognitive states

Cognitive states, including attention, mental workload, mental stress, and mental fatigue, are critical factors in ensuring work quality and safety (Chen et al., 2017). Specifically, cognitive states not only affect construction workers’ health and well-being but also influence their decision-making processes and behavior patterns, thereby impacting work efficiency and safety (Mitropoulos and Memarian, 2012). Although various tools for assessing cognitive states have been developed within academic research, their practical application among construction workers remains limited. This limitation largely stems from the reliance on self-report questionnaires, which, as retrospective feedback tools, have inherent flaws. These tools often lack reliability, are subject to strong subjective biases, and can be influenced by the respondent’s immediate perceptions or emotions, making it difficult to accurately reflect their true cognitive state (Lohani et al., 2019).

The physiological and physical mechanisms related to human cognition are complex, neurons in the brain communicate through electrical and chemical signals (Gligoroska and Manchevska, 2012). When a person is in different cognitive states, such as focusing on a task or being under stress, specific neural circuits are activated. For example, during high-attention tasks, the prefrontal cortex, which is associated with decision-making and attention control, shows increased activity. EEG can capture these electrical signals on the scalp surface, providing a window into these underlying neural activities (Pessoa, 2008). The different frequency bands in EEG, namely *δ*, *α*, *β*, *θ*, and *γ* waves, are related to distinct cognitive functions (Cheng et al., 2022). δ waves are primarily associated with deep sleep. During this stage of sleep, the body undergoes essential restorative processes, and the brain’s activity is at a relatively low level. In some cases of severe brain injury or certain neurological disorders, abnormal delta wave activity may be observed even during wakefulness. α waves are linked to a relaxed yet awake state, often dominant when one is at rest with eyes closed. β waves, associated with active thinking and concentration, prevail during focused mental tasks. θ waves, which emerge during drowsiness, light sleep, and creative thinking, also surface in moments of mental relaxation. γ waves, related to complex cognitive processes like perception and memory integration, play a role in high-level information processing.

EEG is an effective tool for measuring brain activity. By capturing the minute electrical changes on the scalp surface, EEG provides deep insights into the brain’s activity state, especially in evaluating an individual’s cognitive and psychological states (Lan et al., 2005). Since cognitive states are directly related to brain activity, changes in EEG components can intuitively reflect the level of activity within the cognitive system. Consequently, it offers distinct advantages over other physiological indicators, such as eye movement, heart rate, and body temperature, in the assessment of cognitive states. Additionally, its cost-effectiveness, portability, and tolerance to movement make EEG particularly well-suited for use in dynamic construction environments (Sawangjai et al., 2019). By utilizing EEG to detect cognitive states, researchers can address the limitations of existing studies that rely on subjective perceptions of noise effects.

Noise impacts human cognition via multiple pathways (Dohmen et al., 2022). Noise directly stimulates the auditory system, and the resultant signals are transmitted to the brain. This stimulation can disrupt normal neural activity within the auditory cortex and other related brain regions (Sun et al., 2008). For instance, continuous high-intensity noise may cause over-activation of neurons in the auditory cortex, leading to sensory overload. Simultaneously, noise can trigger a stress response in the body. The release of stress hormones, such as cortisol, subsequently affects brain functions, including cognitive processes. Elevated cortisol levels can interfere with the normal communication between neurons in the prefrontal cortex, which is essential for attention and decision-making (McEwen, 2007; Arnsten, 2009). In the case of construction workers, continuous exposure to diverse noise types on construction sites may have cumulative effects on their cognitive states over time.

In recent years, EEG has gained significant attention as a tool for measuring and calculating the cognitive states of construction workers. Researchers have employed EEG to investigate various cognitive states, such as vigilance, mental fatigue, mental stress, attention, mental workload, and emotional states (Cheng et al., 2022). In the research regarding the impact of noise on workers through the utilization of EEG, Ke et al. (2021) directed their focus toward construction workers and employed EEG to explore the manner in which different noise conditions influenced the attention-related brainwave indices of worker subjects, thus furnishing valuable insights into the impact of noise on workers’ attention. Zhang et al. (2019) highlighted the EEG’s potential to capture information about workers’ physical and psychological states during work, suggesting that EEG could effectively enhance safety management and risk control in the construction industry. Within the ambit of research and accomplishments related to EEG, Baig and Kavakli (2018) introduced a novel method to segment EEG data and analyze the correlation between work tasks and cognitive activities. Bai et al. (2017) developed a flight simulation platform based on EEG, opening new possibilities for studying cognitive states in real-world scenarios. Such experimental designs and simulation platform advancements contribute to improving the stability and applicability of EEG in construction safety research. Similarly, Hajinoroozi et al. (2015) extracted features from EEG to predict drivers’ cognitive states, demonstrating the feasibility of applying EEG to analyze construction workers’ cognitive states. Qin et al. (2023) analyzed the impact of EEG technology across various fields, including its potential in construction safety research. Collectively, these studies offer valuable insights and practical approaches that are conducive to the utilization of EEG in investigating the impact of noise on construction workers.

### 2.4 Research gaps in noise impact on workers’ cognitive performance and learning efficiency

The studies reviewed have highlighted the significant emphasis that scholars place on improving the learning and training efficiency of construction workers. The adoption of advanced emerging technologies such as BIM + VR, MR, and VR has been shown to effectively improve training efficiency and quality. Through the creation of immersive and interactive learning environments, these technologies are capable of enhancing the attractiveness and engagement of learning, thereby facilitating the improvement of learning outcomes. Moreover, the research emphasizes the importance of personalized training content. By incorporating the individual differences and characteristics of construction workers, training efficiency and safety performance can be optimized more effectively.

Despite extensive research on learning and training models, methods, and content in the construction industry, the actual working environment, particularly noise, remains a critical factor influencing learning and training efficiency. Noise not only causes hearing loss but also has non-auditory effects on the cardiovascular system, cognitive functions, and psychological well-being, potentially affecting both individual health and work efficiency and safety. However, current research lacks comprehensive and in-depth exploration of how different types and intensities of noise specifically impact workers’ cognitive processes (such as attention, memory, and decision-making) and, subsequently, their learning efficiency. In this context, EEG has emerged as a promising tool for measuring and evaluating construction workers’ cognitive states, with potential applications in safety management and risk control, as it directly measures brain activity to precisely assess cognitive states like vigilance, mental fatigue, and attention, augmenting our understanding of cognitive state influencing factors and providing scientific evidence for safety measures and interventions. Consequently, understanding noise-impact mechanisms and developing effective noise-control measures are crucial for enhancing workplace safety and training efficiency. Building on previous research, this study utilizes EEG to investigate the specific effects of industrial noise on construction workers’ learning efficiency and cognitive states, aiming to identify effective protective measures to mitigate noise-induced harm to their physical and mental health while enhancing learning efficiency. The study’s findings will offer valuable scientific guidance and evidence for improving safety management practices and health-protection strategies in the construction industry.

## 3 Hypotheses and research model

### 3.1 The impact of different types of noise

Hughes et al. (2007) suggested that, compared to complex forms of auditory stimuli, steady auditory stimuli have less impact on the performance of continuous recall tasks. In a review of the literature, Beaman (2005) proposed that complex noise is more disruptive than steady noise, leading to greater auditory distraction and reduced task performance efficiency. Schlittmeier et al. (2012) conducted a modeling analysis of the effects of about 40 types of background sounds on short-term verbal memory, finding that steady sounds have minimal, if any, impact on short-term memory, whereas intermittent and non-steady sounds significantly increase memory error rates. Klatte et al. (2013) also noted that complex noise, such as intermittent or fluctuating noise, tends to be more disruptive as it significantly elevates mental workload and interferes with tasks that require sustained attention and working memory. The unpredictable variations of non-steady noise make it more difficult for individuals to adapt, leading to increased mental fatigue and diminished performance on learning tasks. Based on these findings, the following hypotheses are proposed:

*H1:* Complex noise is more likely to lead to a decrease in learning efficiency compared to steady noise.

*H1a:* Complex noise is more likely to lead to a decrease in accuracy compared to steady noise.

*H1b:* Complex noise is more likely to lead to an increase in reaction time compared to steady noise.

Mir et al. (2022), in their investigation of the effects of different levels and types of construction noise on emotions, found that complex construction noise, such as that produced by saws and handheld drills, more easily affects workers’ cognitive states. In contrast, steady construction noise, such as that produced by bulldozers, is less likely to increase workers’ levels of annoyance and mental stress. Similarly, Lee and Jeon (2013), in their study of the effects of traffic noise, construction noise, and ventilation noise on cognitive performance and subjective perceptions, found that the stability and spectral characteristics of traffic noise, as well as the abrupt fluctuations in construction noise, make these two types of complex noise more annoying compared to the steady noise of ventilation systems. Moreover, the cognitive impact of these noises on cognitive tasks varies significantly depending on their characteristics. Lee et al. (2019) further evaluated the effects of noise generated by different machines on construction workers, revealing that complex noise produced by machines such as crushers, pile drivers, and hammer compaction machines has the most substantial impact on workers’ cognitive states. Based on these findings, the following hypotheses are proposed:

*H2:* Complex noise is more likely to lead to poorer cognitive states compared to steady noise.

*H2c:* Complex noise is more likely to reduce attention compared to steady noise.

*H2d:* Complex noise is more likely to increase mental workload compared to steady noise.

*H2e:* Complex noise is more likely to increase mental fatigue compared to steady noise.

*H2f:* Complex noise is more likely increase mental stress compared to steady noise.

### 3.2 The impact of different noise levels

Xiong et al. (2018) demonstrated that the level of construction noise significantly affects learning efficiency. When noise levels exceed 55 dB(A), physiological reactions such as dizziness and emotional discomfort may occur, leading to communication disruptions and decreased academic performance. In an experiment by Ke et al. (2021) investigating the effects of noise on workers’ hazard recognition, it was found that higher noise levels reduced the accuracy of recognition and increased reaction time. Fernandes et al. (2019) examined the effects of two different noise levels on students’ performance during reading and writing tasks. Their results showed that higher noise levels led to decreased attention and accuracy. Based on these findings, the following hypotheses are proposed:

*H3:* Higher noise levels are more likely to lead to a decrease in learning efficiency.

*H3a:* Higher noise levels are more likely to lead to a decrease in accuracy.

*H3b:* Higher noise levels are more likely to lead to an increase in reaction time.

Sharma et al. (2019) conducted an empirical study exploring the impact of noise type and noise level on human cognitive performance. The results showed that cognitive performance was significantly affected by both noise type and noise level. Noise type negatively influenced reaction time, while higher noise levels substantially increased errors during cognitive tasks. Cognitive abilities deteriorated significantly when noise levels reached 85 dB(A) across all types of noise. Jafari et al. (2019) evaluated participants’ mental workload and attention under background noise, and noise levels of 75 dB(A), 85 dB(A), and 95 dB(A). The findings indicated that at 95 dB(A), mental workload and attention significantly decreased, whereas the effects were not significant at 75 dB(A) and 85 dB(A). In a study by Kordmiri et al. (2023), it was found that high noise levels significantly increased workers’ mental fatigue. Based on these findings, the following hypotheses are proposed:

*H4:* Higher noise levels are more likely to lead to poorer cognitive states.

*H4c:* Higher noise levels are more likely to lead to a decrease in attention.

*H4d:* Higher noise levels are more likely to lead to an increase in mental workload.

*H4e:* Higher noise levels are more likely to lead to an increase in mental fatigue.

*H4f:* Higher noise levels are more likely to lead to an increase in mental stress.

**Figure 1:**
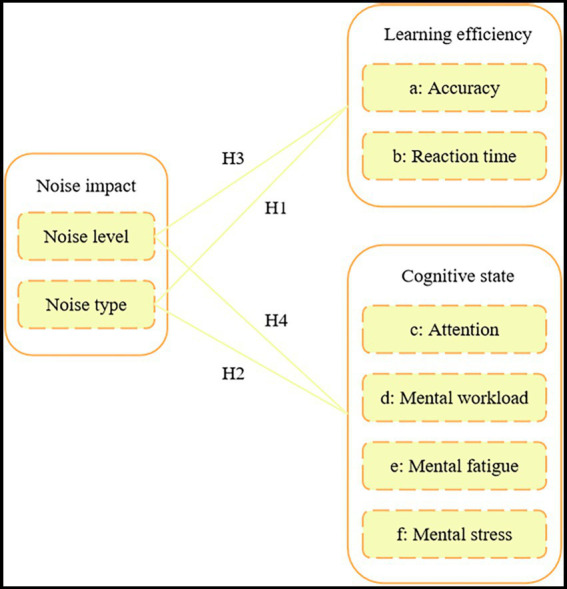
Theoretical model framework diagram.

The theoretical model framework based on these research hypotheses is illustrated in Figure 1.

## 4 Methods

### 4.1 Experimental instruments and materials

#### 4.1.1 Experimental instruments

The instruments used in this study are primarily for measuring environmental noise and collecting EEG signals. According to the “Environmental Noise Emission Standards for Construction Site Boundaries” (GB12523-2011), noise measurement instruments must be automatic noise monitors or integrating sound level meters, and their performance should meet the requirements specified in the “Integrating Sound Level Meters” (GB/T17181). Therefore, this study uses the Shima AS824, a professional sound level meter that adheres to these standards, to accurately measure noise levels. The Emotiv-EPOC X EEG device was selected to capture EEG signals from participants exposed to varying noise environments. This device is equipped with 14 high-sensitivity electrode channels that cover key brain regions, including the frontal lobe (AF3, AF4, F3, F4, F7, F8, FC5, FC6), temporal lobe (T7, T8), parietal lobe (P7, P8), and occipital lobe (O1, O2). Electrode placement follows the international 10–20 system, a widely recognized standard for EEG electrode placement, ensuring precision, consistency, and comparability in electrode positioning.

#### 4.1.2 Noise materials

To simulate the noise conditions that construction workers face in real working environments and to investigate the impact of different types and levels of noise on workers’ learning efficiency, noise samples were directly collected from a component factory’s production site. These noise samples cover common noise sources found on construction sites, such as the continuous operation of air compressors and the drilling sounds from electric drills, accurately reflecting the actual noise environment of construction workers’ daily activities. Sound editing software was used to isolate the main noise sources from the original recordings, and the noise was classified into two groups based on industrial noise types: the steady noise group and the complex noise group. The steady noise group consists primarily of continuous and relatively uniform noise produced by sources like air compressors and electric drills, characterized by probability density functions that follow a Gaussian distribution. In contrast, the complex noise group is based on the steady noise group but includes transient high-energy impulse noises, such as hammering, which introduce sudden, high-intensity sounds, making the overall noise environment more complex and unpredictable.

To more accurately study the effect of noise intensity on workers’ learning efficiency, it should be noted that both the steady noise group and the complex noise group have their own dedicated control groups. This setup facilitates better accounting for various factors and isolating the impact of different noise types. Each of these two noise groups, namely the steady noise group and the complex noise group, was further divided into subgroups according to intensity levels: the control group, low noise group, medium noise group, and high noise group. For the control groups corresponding to both the steady noise group and the complex noise group, white noise from workers’ daily lives was used as a baseline condition for comparison. The low noise groups of both the steady and complex noise groups had average decibel values set at 60 dB(A), the medium noise groups at 70 dB(A), and the high noise groups at 80 dB(A). This approach enables a comprehensive analysis of how different noise intensities within each noise type affect workers’ learning efficiency.

#### 4.1.3 Learning task materials

A teaching and testing program was designed based on the principle of assessing construction workers’ learning efficiency under different noise environments. The teaching materials and test questions used in the experiment were sourced from the official question bank of the “National Prefabricated Construction Vocational Skills Competition,” ensuring the professionalism and practical value of the content. To better align with the specific learning needs and practical experience of the workers, a training instructor from the component factory, with extensive teaching experience, was invited to participate in the creation of the instructional videos.

To ensure fairness and scientific integrity, four instructional videos were produced, each providing a concise and detailed explanation of specific knowledge points. The videos were designed with high information density to accommodate the unique demands of learning in noisy environments. To test learning outcomes, 20 test questions were prepared for each video. These questions were directly based on the video content and aimed to assess the viewers’ understanding and mastery of the instructional material.

During the design process of the teaching content and test questions, endeavors were made to keep the difficulty levels similar across all videos and their corresponding tests, thereby ensuring the consistency and comparability of the experimental results. Moreover, the difference in text length between the teaching content and test questions for each video was controlled within 10%. This measure was adopted to minimize variations in the learning burden induced by differences in content lengths, ensuring that the experimental results could more accurately reflect the impact of noisy environments on learning efficiency.

### 4.2 Experimental participants

This study recruited 22 construction workers as experimental participants, evenly divided into two groups: 11 in the complex noise group and 11 in the steady noise group. Each group was composed of 9 men and 2 women. This gender-balanced distribution within the groups helps to minimize potential confounding factors related to gender in the analysis of the impact of different noise types on the participants. The ages of the participants in both groups spanned from 18 to 50 years, with an average age of 35 years. The majority of them were between 25 and 45 years old. Regarding work experience, it ranged from 1 to 30 years, with an average of 12 years. A significant proportion of the participants had 1–10 years of experience, while some had more than 15 years of experience. The experimental protocol was approved by the Ethics Committee of Hainan University. The participants represented a diverse range of ages, from younger workers to more seasoned professionals, thus reflecting the actual demographic distribution within the construction industry’s workforce. All participants had normal vision and hearing, normal noise tolerance, and were capable of text recognition and verbal communication. They were instructed to avoid alcohol or stimulants prior to the experiment and actively participated in completing the experimental tasks throughout the study.

### 4.3 Data collection

In this experiment, E-Prime 3.0 software was used to collect behavioral data from the participants. After confirming that all experimental equipment and software were properly set up and calibrated, the E-Prime 3.0 software was launched, and the pre-designed experimental program was loaded. This program included the playback of instructional videos, the answering tasks, and the randomization of noise conditions. Once the experimental program was initiated, E-Prime 3.0 automatically guided the participants through the entire experiment, including watching instructional videos, completing multiple-choice questions, and taking breaks. During each response phase, the software tracked both the participants’ answers and their reaction times. Throughout the entire experiment, E-Prime 3.0 continuously and automatically recorded data on the accuracy of responses and reaction times, ensuring comprehensive data collection. After the experiment, the data files were checked to ensure that all information was correctly saved, and the collected data were exported for subsequent statistical analysis.

EEG data were collected using the Emotiv-EPOC X device during this experiment. Before the experiment began, the Emotiv-EPOC X device and its accessories were checked for any damage, and the accompanying recording software was properly installed. The electrodes were coated with an appropriate amount of conductive gel to ensure good signal transmission. Prior to the start of the experiment, participants were guided through proper scalp cleaning procedures, after which EEG electrodes were applied according to the international 10–20 system. Care was taken to ensure that each electrode maintained secure contact with the scalp for accurate data collection. The electrode cap was adjusted to fit comfortably on each participant’s head. Once the electrodes were securely placed, the EEG recording software was initiated to assess signal quality, ensuring that all channels were functioning within acceptable ranges. If any channel showed poor signal quality, adjustments were made to the electrode positions, or additional conductive gel was applied. As participants began the learning tasks, the EEG device was activated to start real-time recording of EEG data. Throughout the experiment, the EEG signal quality was continuously monitored to maintain both accuracy and completeness of the data. After the experiment, all EEG data were securely stored, with each data set properly labeled for subsequent analysis.

### 4.4 Experimental procedure

During the experimental preparation phase, participants’ demographic information—including age, gender, and educational background—was systematically recorded. The experimental procedures and task requirements were thoroughly explained to the participants, and they were provided with sufficient practice sessions to familiarize themselves with the protocol. This preparatory phase aimed to ensure that participants fully understood the purpose of the experiment and reduce potential confusion or anxiety. Prior to the placement of electrodes, the participants’ scalps were meticulously cleaned to eliminate any contaminants such as dirt and oils, thereby optimizing electrode-skin contact to enhance signal quality. Following the electrode installation, their connections were rigorously checked to ensure proper attachment, and the correct positioning of the reference and ground electrodes was verified. Signal quality was assessed to confirm the proper functionality of the EEG recording software. Additionally, sound levels were calibrated using noise measurement instrument to ensure that the noise conditions conformed to the predefined experimental standards.

**Figure 2:**
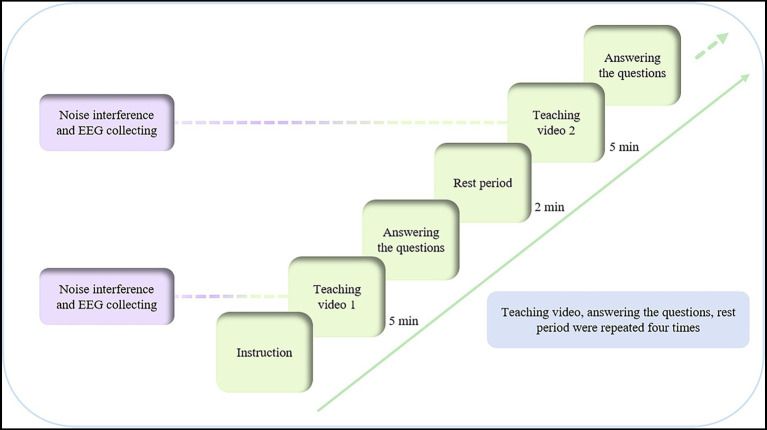
Experimental procedure flowchart.

During the formal experimental phase, each participant, after thoroughly reviewing and confirming their understanding of the experimental instructions, initiated the procedure by pressing the start button. Upon commencement, the participant watched a five-minute teaching video. During this video-watching period, noise interference was introduced, and the EEG signals were continuously recorded. Note that the noise was present only when the participants were watching the teaching video. Following the video, the participant completed 20 multiple-choice questions directly related to the video content, with a 20-s time limit allocated for each question. Upon task completion, participants were provided with a two-minute rest period to alleviate fatigue and prepare for the subsequent task. This procedure was repeated four times, with each cycle maintaining consistency in steps, while the noise levels and teaching videos were randomly assigned and shuffled to introduce variability. A comprehensive outline of the experimental protocol is presented in Figure 2.

### 4.5 Data analysis

During the entire EEG recording process, the collected data are highly vulnerable to various artifacts, which can significantly affect data quality and complicate subsequent data processing and analysis. Therefore, EEG data preprocessing is essential to minimize the impact of these artifacts. Artifacts are generally classified into two categories: intrinsic and extrinsic. Intrinsic artifacts primarily originate from the participant’s physiological activities, such as eye blinks and heartbeats, which produce noticeable distortions in the EEG signals. Extrinsic artifacts, on the other hand, stem from the experimental environment and equipment, such as electrode displacement due to head movement or loose connections, which can compromise the quality of the EEG recordings.

**Figure 3:**
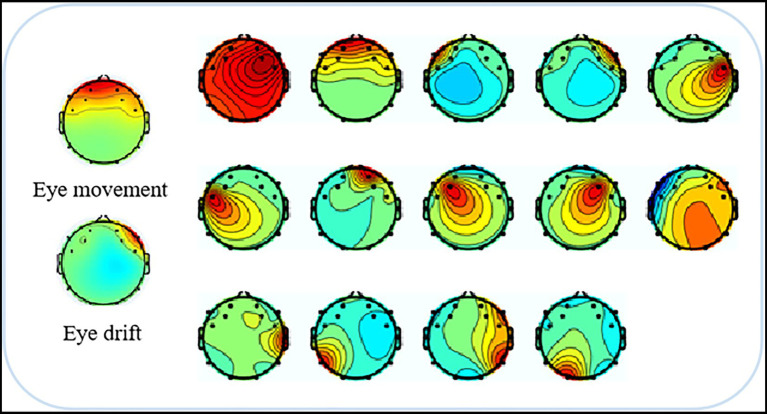
Removal of eye movement and drift artifacts from EEG signals using ICA analysis.

**Figure 4:**
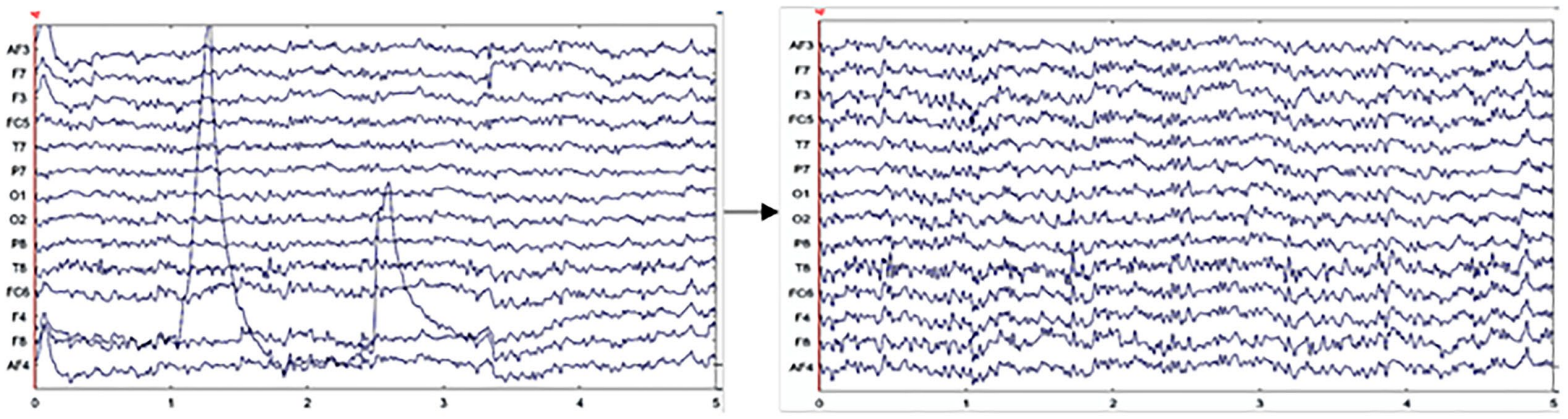
Comparison of EEG signals before and after artifact removal.

To minimize the impact of artifacts and obtain clean, valid EEG data, the EEG signal processing framework proposed by Jebelli et al. (2018) was followed, and the EEG data were preprocessed according to the steps outlined. First, the raw EEG data were imported into the signal processing software EEGLAB. Upon importing the data, electrode location information was used to verify the precise positioning of each electrode, ensuring accuracy for subsequent analysis. Subsequently, a Finite Impulse Response (FIR) filter was applied to eliminate the majority of extrinsic artifacts, including electrode movement and power line interference, along with low-frequency (below 0.5 Hz) and high-frequency (above 40 Hz) interference induced by physiological activities like respiration and muscle movements, among which heartbeats were included. After filtering, interpolation was used to correct any faulty electrodes, and bad data segments were removed from the data set. Finally, Independent Component Analysis (ICA) was performed to eliminate artifacts. As shown in Figure 3, most intrinsic artifacts caused by muscle movements, such as eye movements and eye drifts, can be isolated into independent components through ICA. These artifact components were then identified and removed. After preprocessing, clean EEG data were obtained, as illustrated in Figure 4.

**Figure 5:**
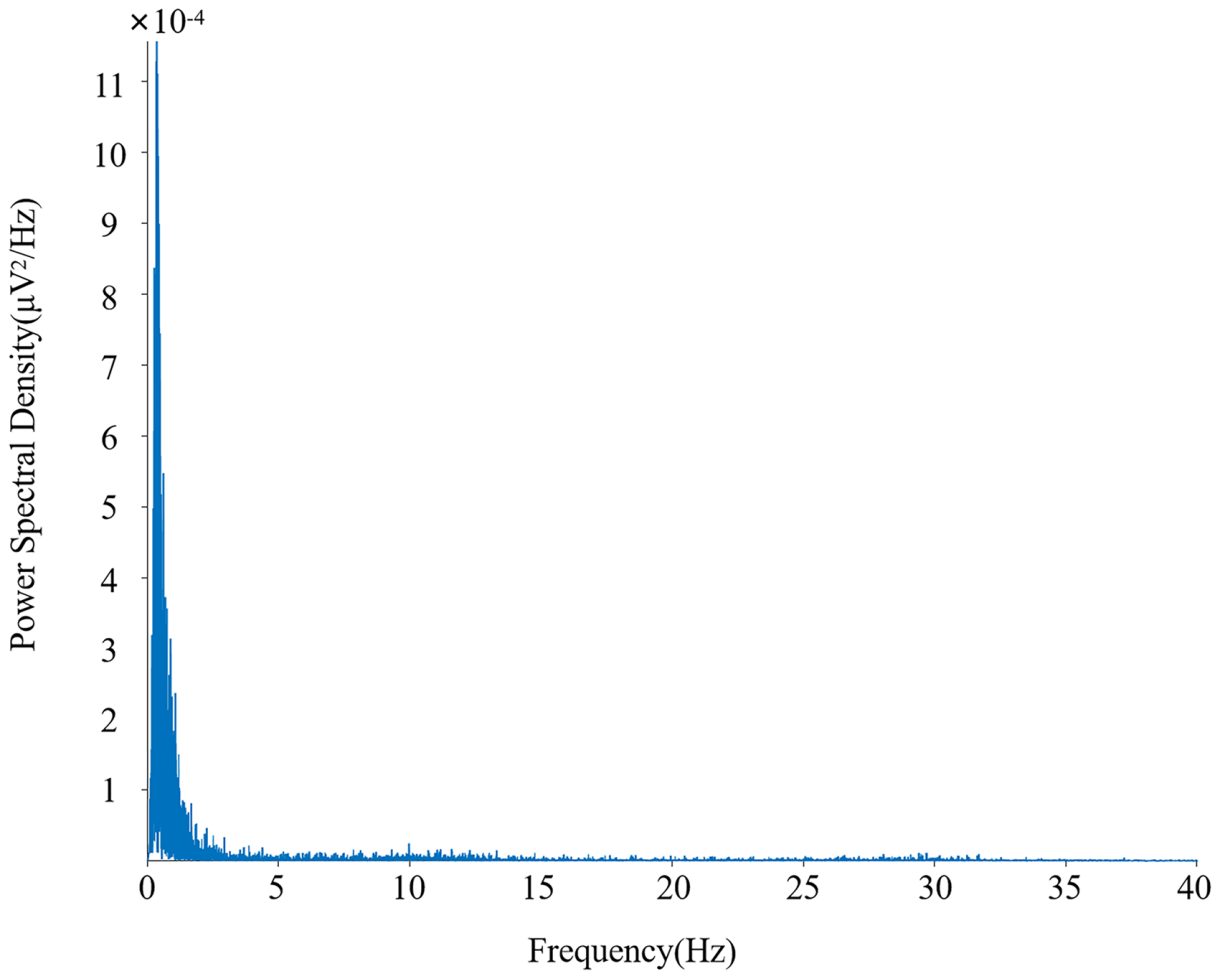
Electroencephalogram power spectral density plot.

EEG signals can be categorized into five fundamental frequency bands, namely *δ* (1–4 Hz), *θ* (5–7 Hz), *α* (8–14 Hz), *β* (15–30 Hz), and *γ* (above 30 Hz). Power Spectral Density (PSD), which is derived by utilizing MATLAB’s pwelch function, is employed to quantify the relative PSD values within these bands, thereby providing a basis for the assessment of cognitive states including attention, mental workload, mental fatigue, and mental stress (Cheng et al., 2022). The detailed calculation formulas for these indicators are presented in Table 1. The specific computational parameters are set as follows: The sampling rate is set at 128 Hz. A Hamming window is employed for smoothing purposes. The window length is determined to be 2 s, which correspondingly contains 256 sampling points. Additionally, a 50% overlapping window ratio is utilized to optimize the accuracy and reliability of the data analysis. Figure 5 presents a clear depiction of the PSD of the EEG signal. The curve represents the PSD values, which exhibit a prominent peak at lower frequencies, gradually decreasing as the frequency increases. This characteristic pattern is typical in EEG power spectra, where lower frequencies often carry more significant power. This figure effectively illustrates the broadband power spectrum of EEG obtained using the specified parameters, providing a visual representation of the frequency-domain characteristics of the EEG data and supporting the calculation of cognitive states.

**Table 1:** Calculation formulas for cognitive state indicators.

Cognitive state	Calculation formula	Energy source channels
Attention	Arousal index = *α*_i_/*β*_i_	i = AF3 + AF4 + F4 + F3
Mental workload	Engagement index = β/(θ + α)	Average of all channels
Mental fatigue	θ/α	Average of all channels
Mental stress	β_i_	i = T7/T8/AF3/AF4

## 5 Results

### 5.1 Behavioral data analysis

**Figure 6:**
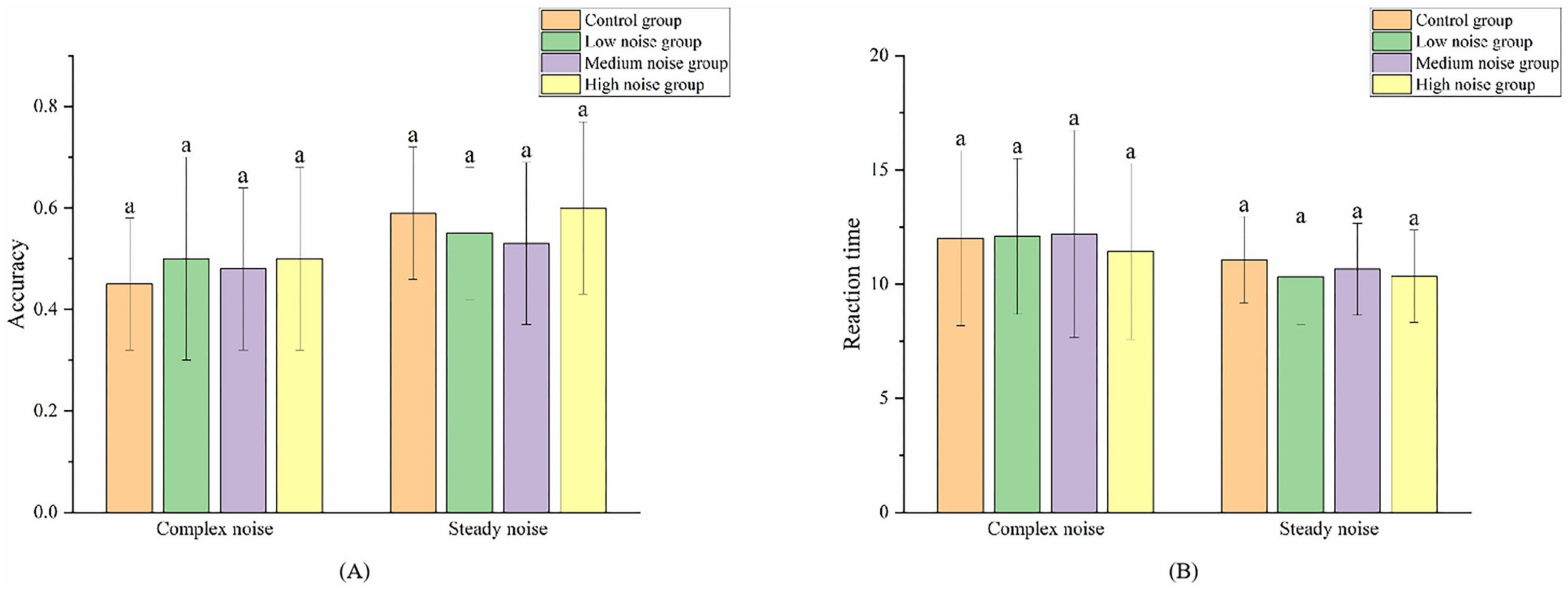
Comparison of accuracy **(A)** and reaction time **(B)** across different groups.

The accuracy and reaction time of participants in both the complex noise and steady noise groups are shown in Figure 6. Overall, the accuracy at all noise levels in the complex noise environment is lower than that in the steady noise environment. This suggests that the complex noise environment more easily impairs workers’ ability to comprehend and correctly apply the knowledge they have learned compared to the steady noise environment. The most pronounced difference occurs in the high noise group, indicating that high levels of complex noise have a greater adverse impact on comprehension and application of knowledge than equivalent levels of steady noise environment. Similarly, reaction time across all noise levels are longer in the complex noise environment than in the steady noise environment. These findings indicates that, compared to the steady noise environment, a complex noise environment more readily increases workers’ reaction times. This effect is particularly noticeable in the low noise group and the medium noise group, suggesting that low and medium noise levels in a complex environment have a greater impact on workers’ familiarity with and ability to quickly apply knowledge than equivalent levels in a steady noise environment.

A significance analysis was conducted on the differences in accuracy and reaction time across various noise levels in the two noise environments, as shown in Table 2. The results revealed no significant differences in the impact of different noise levels on accuracy and reaction time within each noise environment. This indicates that the type of noise has a greater effect on workers’ learning efficiency, whereas variations in noise levels within the same noise type are not significantly impactful.

**Table 2:** Kruskal-Wallis ANOVA results on the impact of noise levels on behavioral data.

Noise type	Factor	Behavioral indicator	Degrees of freedom	*H*	*p*
Complex noise	Noise level	Accuracy	3	0.446	0.931 > 0.05
		Reaction time	3	2.235	0.525 > 0.05
Steady noise		Accuracy	3	0.333	0.954 > 0.05
		Reaction time	3	1.294	0.731 > 0.05

### 5.2 Cognitive state analysis

#### 5.2.1 Analysis of the impact of noise on attention

**Figure 7:**
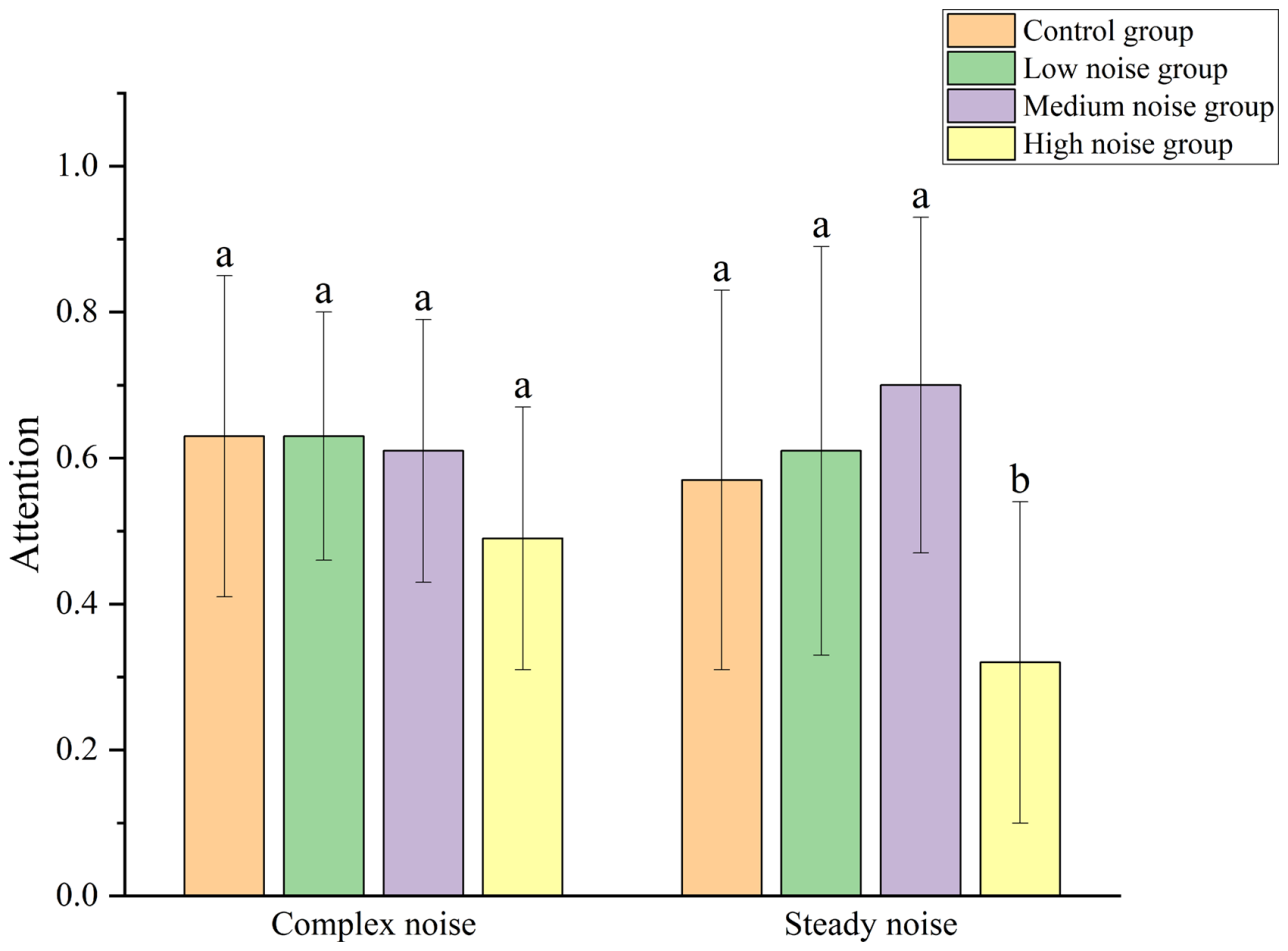
Comparison of attention across different groups.

As depicted in Figure 7, the impact of complex versus steady noise on attention reveals differing response patterns between the two environments. Specifically, under complex noise conditions, both the low and high noise groups exhibited better attention performance than their counterparts in the steady noise environment. In contrast, the medium noise group in the complex noise environment showed lower attention performance compared to the medium noise group in the steady noise environment. Overall, the type of noise did not exhibit a consistent pattern of influence on attention.

A Mann–Whitney U test was conducted to further compare attention levels under the two types of noise environments. The results indicated that there was no statistically significant difference in attention levels between the two noise environments (Z = -0.643, *p* > 0.05), suggesting that different noise types do not have a significant impact on attention levels.

Additionally, a separate statistical analysis was performed on the attention levels across different noise intensity levels within each noise type, and the results are presented in Table 3. In the complex noise environment, there were no significant differences in attention indicators across the different noise levels. However, in the steady noise environment, different noise levels had a significant impact on attention, with a relatively large effect size. This indicates that noise level in this environment has a strong influence on attention.

**Table 3:** Kruskal-Wallis ANOVA results on the impact of noise levels on attention.

Noise type	Factor	EEG indicator	Degrees of freedom	*H*	*p*	*η* ^2^
Complex noise	Noise level	Attention	3	3.68	0.298 > 0.05	-
Steady noise			3	10.64	0.014 < 0.05	0.248

To gain deeper insights into the inter-group relationships of attention across different noise levels within the steady noise environment, a post-hoc multiple comparison was conducted. The results, as presented in Table 4, reveal that the attention level at 80 dB (A) is significantly lower than that at the other three noise levels [i.e., the control group, 60 dB (A), and 70 dB (A)], with a relatively strong effect, whereas no significant differences in attention exist among the other three noise levels.

**Table 4:** Post-hoc multiple comparison of attention across different noise levels in the steady noise environment.

Comparison groups	Test statistic	Standard error	*p*	Cohen’s *d*	Result
Control group vs. High noise group	11.364	5.477	0.038 < 0.05	1.723	High noise group < Control group
High noise group vs. Low noise group	12.818	5.477	0.019 < 0.05	2.000	High noise group < Low noise group
Medium noise group vs. High noise group	17.091	5.477	0.002 < 0.05	2.494	High noise group < Medium noise group
Control group vs. Low noise group	−1.455	5.477	0.791 > 0.05	-	No significant difference
Control group vs. Medium noise group	−5.727	5.477	0.296 > 0.05	-	No significant difference
Medium noise group vs. Low noise group	−4.273	5.477	0.435 > 0.05	-	No significant difference

The results indicate that in a steady noise environment, significant negative effects on workers’ attention occur only when the noise level reaches or exceeds 80 dB(A). At this threshold, the disruptive effect of noise on individuals’ cognitive function and work efficiency sharply increases. In contrast, noise levels below this threshold have a relatively minor effect on attention, suggesting that individuals can partially adapt to or ignore lower levels of noise, thereby maintaining better work performance and attentional focus.

#### 5.2.2 Analysis of the impact of noise on mental workload

**Figure 8:**
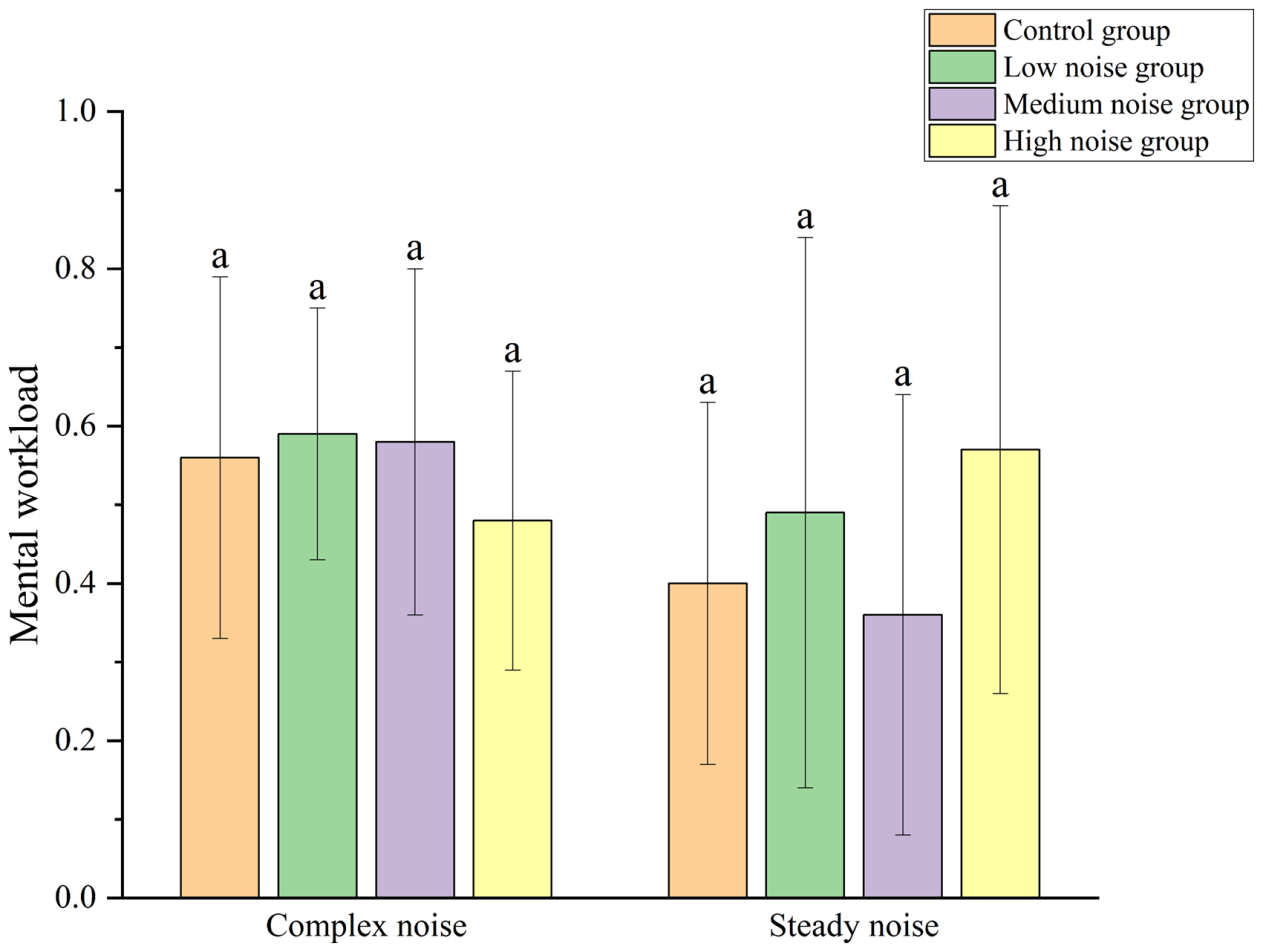
Comparison of mental workload across different groups.

The impact of complex noise and steady noise on mental workload is illustrated in Figure 8. The results indicate that mental workload levels do not exhibit a consistent trend across the same noise levels. Specifically, in the low and medium noise groups, participants experienced higher mental workload under complex noise conditions compared to steady noise conditions. However, in the high noise group, the mental workload under complex noise is lower than that under steady noise.

A Mann–Whitney U test was conducted to compare mental workload levels under the two types of noise environments. The results indicated no statistically significant difference between the complex noise and steady noise conditions (Z = -1.719, *p* > 0.05), suggesting that the type of noise does not significantly affect mental workload levels.

Furthermore, a statistical analysis of mental workload across different noise levels within each noise environment was performed. The analysis revealed no statistically significant impact of different noise levels on mental workload in either noise environment (Complex noise: p > 0.05, Steady noise: p > 0.05). This indicates that, within the same noise type, different noise intensities do not have a significant differential impact on workers’ mental workload.

Statistical analysis indicates that variations in noise intensity within the same noise type do not significantly affect workers’ mental workload levels. This suggests that workers maintain relative stability in their cognitive processing ability and the psychological stress during task execution when exposed to different noise intensities. It implies that workers possess a certain level of adaptability to their noise environment, and the differences between noise levels are insufficient to impact mental workload.

#### 5.2.3 Analysis of the impact of noise on mental fatigue

**Figure 9:**
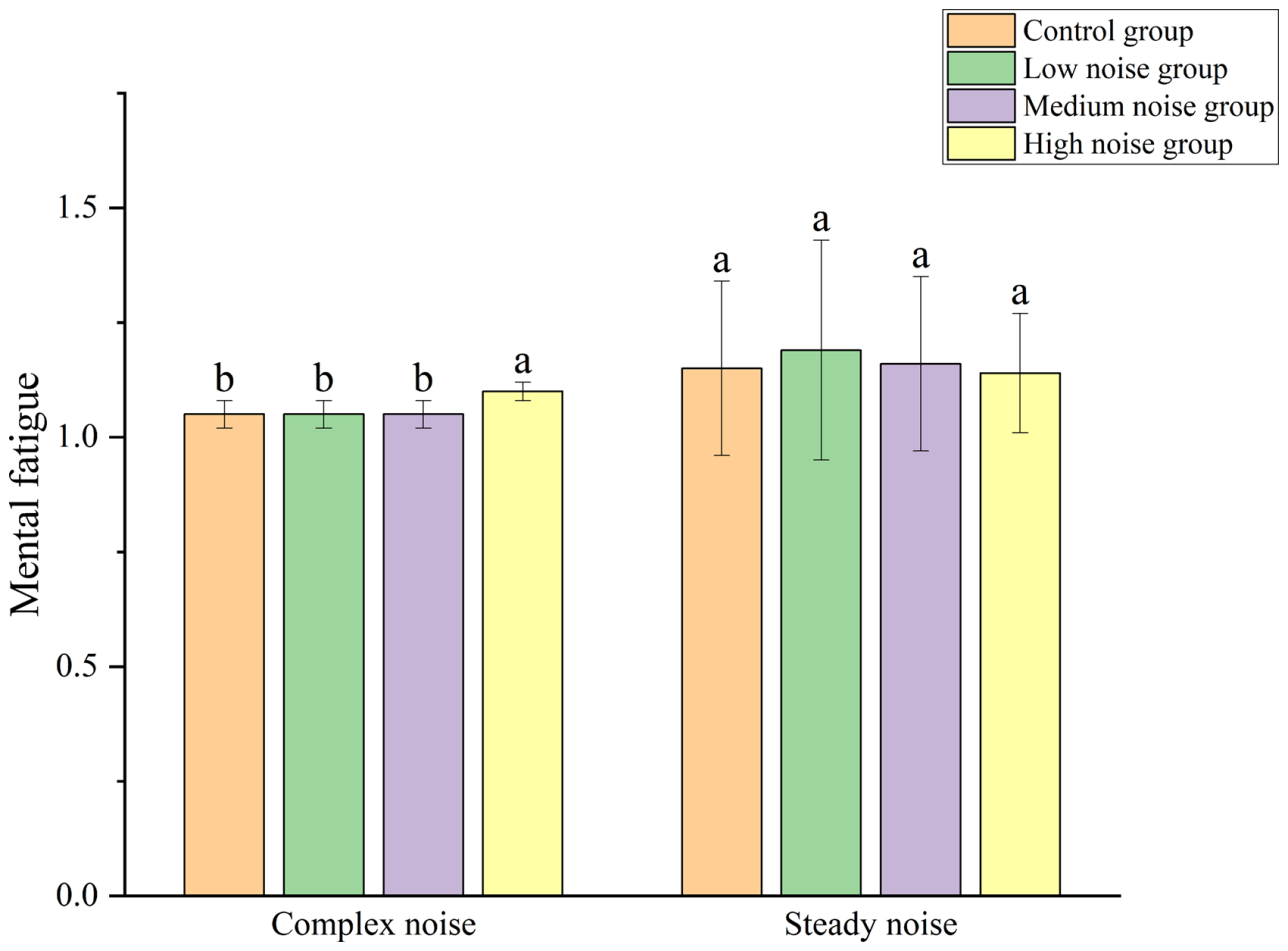
Comparison of mental fatigue across different groups.

Figure 9 depicts the impact of complex noise versus steady noise on mental fatigue. The data demonstrate that mental fatigue levels are consistently higher across all noise intensities within the steady noise environment in comparison with the complex noise environment. This suggests that, compared to a complex noise environment, a steady noise environment is more likely to induce mental fatigue in workers.

A statistical analysis was conducted on mental fatigue across different noise intensity levels within each noise type, and the results are presented in Table 5. In the steady noise environment, no significant differences in mental fatigue were observed across varying noise levels, indicating that noise intensity did not substantially affect mental fatigue under steady noise conditions. Conversely, under complex noise conditions, different noise levels had a significant impact on mental fatigue, with a relatively strong effect size, highlighting the notable role of noise levels in modulating mental fatigue.

**Table 5:** Kruskal-Wallis ANOVA results on the impact of noise levels on mental fatigue.

Noise type	Factor	EEG indicator	Degrees of freedom	H	*p*	η^2^
Complex noise	Noise level	Mental fatigue	3	19.986	<0.01	0.465
Steady noise			3	0.239	0.971 > 0.05	-

A post-hoc multiple comparison of mental fatigue across different noise levels within the complex noise environment was conducted, and the results are presented in Table 6. In the complex noise environment, the high noise group resulted in significantly higher mental fatigue compared to the medium noise group and the low noise group, exhibiting an extremely strong effect. However, there was no significant difference in mental fatigue between the high noise group and the control group. Additionally, no significant differences were found in the comparisons among the other three groups. This indicates that the 80 dB(A) noise level in a complex noise environment leads to a significant increase in mental fatigue.

**Table 6:** Post-hoc multiple comparison of mental fatigue across different noise levels in the complex noise environment.

Comparison groups	Test statistic	Standard error	*p*	Cohen’s d	Result
Control group vs. High noise group	−14.455	5.477	0.146 > 0.05	-	No significant difference
High noise group vs. Low noise group	−24.273	5.477	0.004 < 0.05	2.982	High noise group > Low noise group
Medium noise group vs. High noise group	−22.727	5.477	0.009 < 0.05	3.042	High noise group > Medium noise group
Control group vs. Low noise group	9.818	5.477	0.97 > 0.05	-	No significant difference
Control group vs. Medium noise group	8.273	5.477	1.000 > 0.05	-	No significant difference
Medium noise group vs. Low noise group	−1.545	5.477	1.000 > 0.05	-	No significant difference

The research results indicate that steady noise is more likely than complex noise to induce higher levels of mental fatigue in workers. Moreover, when the intensity of complex noise rises to 80 dB(A), there is a significant rise in workers’ mental fatigue. This suggests that the complexity and variability of noise can, to some extent, mitigate the negative impact of noise on mental fatigue. However, noise intensity remains a critical factor, once it reaches a certain threshold, it significantly elevates workers’ mental fatigue regardless of its complexity.

#### 5.2.4 Analysis of the impact of noise on mental stress

**Figure 10:**
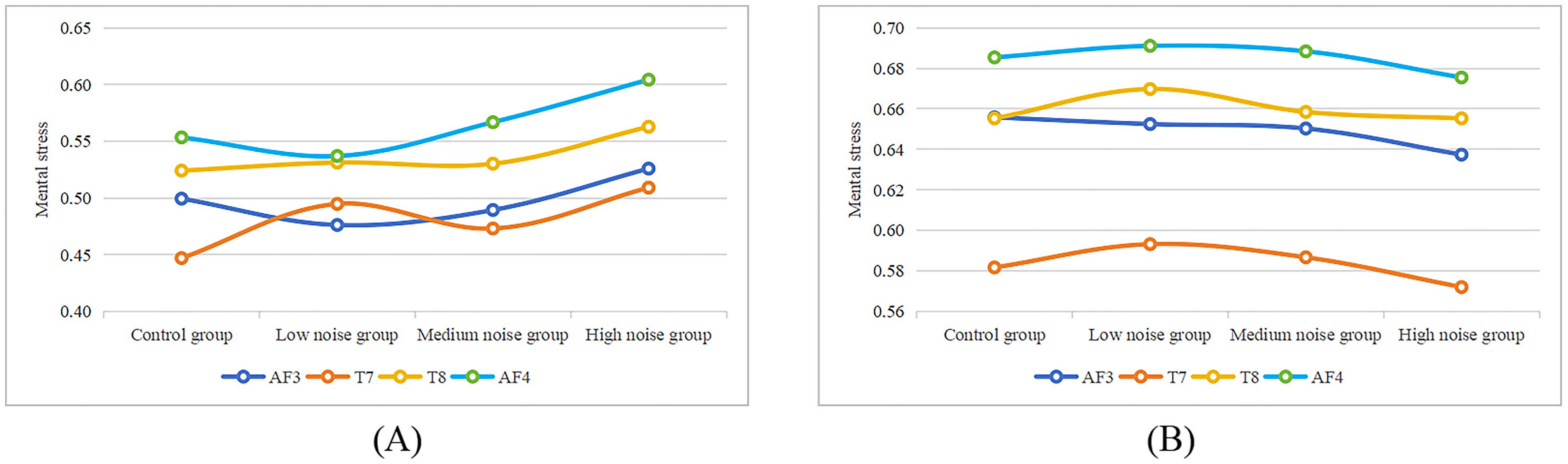
Mental stress across different noise levels in the steady noise group **(A)** and complex noise group **(B)** for each channel.

The impact of steady noise and complex noise on mental stress is illustrated in Figure 10. The prefrontal cortex (AF3, AF4) and temporal lobe regions (T7, T8) are the areas associated with mental stress. Mental stress levels under complex noise were higher than those under steady noise at the same noise levels, indicating that complex noise is more likely to increase mental stress compared to steady noise.

In order to further analyze the impact of different noise levels on mental fatigue within the two noise environments, a Kruskal-Wallis ANOVA was carried out. The results demonstrated that there was no statistically significant effect of varying noise levels on mental fatigue in either the steady or complex noise environments (Complex noise: *p* > 0.05, Steady noise: p > 0.05). This implies that, within a certain range, changes in noise intensity do not exert a significant impact on workers’ mental fatigue.

Further observation revealed that, in the steady noise environment, mental fatigue tended to increase as noise levels escalated. Although this trend did not reach statistical significance, it implies that exposure to steady noise might potentially aggravate workers’ mental fatigue.

### 5.3 Correlation analysis between cognitive state indicators and behavioral indicators

To further investigate the relationships and internal correlations between behavioral indicators and cognitive state indicators, this study employed the Pearson correlation coefficient (r) as the primary tool for measuring associations between variables. The correlation results are presented in Table 7. A negative correlation was found between reaction time and attention, indicating higher levels of attention are associated with shorter reaction times. This finding supports the hypothesis that increased attention can effectively enhance processing speed. Additionally, a significant positive correlation was observed between reaction time and mental workload, suggesting that as mental workload increases, reaction time also increases. This implies that an individual’s response speed decreases with higher mental workload, possibly due to reduced information processing efficiency caused by excessive mental workload.

**Table 7:** Correlation coefficients between behavioral indicators and cognitive state indicators.

Correlation indicators	*r*	*p*
Accuracy - Attention	0.009	0.931 > 0.05
Accuracy - Mental workload	0.155	0.150 > 0.05
Accuracy - Mental stress	0.118	0.275 > 0.05
Accuracy - Mental fatigue	−0.017	0.877 > 0.05
Reaction time - Attention	−0.220	0.039 < 0.05
Reaction time - Mental workload	0.267	0.012 < 0.05
Reaction time - Mental stress	0.229	0.032 < 0.05
Reaction time - Mental fatigue	−0.161	0.133 > 0.05

A positive correlation was also found between reaction time and mental stress, suggesting that higher mental stress may impair an individual’s information processing ability and response speed. However, the correlation between reaction time and mental fatigue was not significant, revealing that mental fatigue does not directly affect reaction time or that its impact may be too subtle to detect statistically. Regarding accuracy, no significant correlations were found between accuracy and any of the cognitive state indicators. This suggest that accuracy is influenced by multiple factors, and no single cognitive state indicator can fully explain its variation.

## 6 Discussion

Based on the experimental results, the hypotheses were systematically tested, and the outcomes are presented in Table 8. The following section provides a detailed discussion and analysis of the results for each individual hypothesis test.

**Table 8:** Hypothesis test results.

Hypothesis	Result
*H1a:* Complex noise is more likely to lead to a decrease in accuracy compared to steady noise.	Supported
*H1b:* Complex noise is more likely to lead to an increase in reaction time compared to steady noise.	Supported
*H2c:* Complex noise is more likely to lead to a decrease in attention compared to steady noise.	Not supported
*H2d:* Complex noise is more likely to lead to an increase in mental workload compared to steady noise.	Not supported
*H2e:* Complex noise is more likely to lead to an increase in mental fatigue compared to steady noise.	Not supported
*H2f:* Complex noise is more likely to lead to an increase in mental stress compared to steady noise.	Supported
*H3a:* Higher noise levels are more likely to lead to a decrease in accuracy.	Not supported
*H3b:* Higher noise levels are more likely to lead to an increase in reaction time.	Not supported
*H4c:* Higher noise levels are more likely to lead to a decrease in attention.	Supported
*H4d:* Higher noise levels are more likely to lead to an increase in mental workload.	Not supported
*H4e:* Higher noise levels are more likely to lead to an increase in mental fatigue.	Supported
*H4f:* Higher noise levels are more likely to lead to an increase in mental stress.	Supported

### 6.1 The impact of noise type on learning efficiency and cognitive state

The experimental results lent support to hypothesis H1, which posited that at the same noise level, complex noise exerted a significantly more negative impact on construction workers’ learning efficiency than did steady noise. Specifically, under identical noise levels, complex noise was demonstrated to have a more pronounced negative influence on the learning efficiency of construction workers in comparison with steady noise. The unpredictability and frequent variations of complex noise made it challenging for workers to adapt and ignore, leading to constant disruption of their working memory and information processing capabilities. In a complex noise environment, workers had to frequently reallocate and adjust their attention, leading to increased mental exhaustion (Hughes et al., 2007). High levels of mental workload and prolonged mental stress not only reduced learning accuracy and reaction speed but also further affected workers’ overall learning efficiency and job performance (Klatte et al., 2013). Additionally, the diversity and variability of complex noise continuously captured workers’ attention, disrupting their workflow and increasing the strain on their cognitive resources (Beaman, 2005). In contrast, while steady noise did affect cognitive function to some degree, its consistent nature allowed workers to habituate and adapt more easily, thereby reducing interference with learning tasks (Schlittmeier et al., 2012). On the other hand, the intermittent and irregular nature of complex noise increased the mental workload, making workers more susceptible to mental fatigue and mental stress, which further impaired their learning outcomes and work efficiency.

Regarding the impact of noise type on cognitive state, the experimental results did not support hypotheses H2c and H2d. This outcome aligns with the findings of Sherman et al. (2023) and Li et al. (2023), where the effect of noise type on attention and mental workload did not reach statistical significance. One possible explanation is that individuals adapted to different noise types, reducing their interference of noise on attention and mental workload. Additionally, there may be a threshold beyond which noise must exceed a certain intensity or duration to significantly affect cognitive performance (Lavie and Polly, 2014). More specifically, workers’ adaptation to continuous noise likely involves several cognitive and physiological processes. Habituation may occur as the auditory cortex and related neural pathways adjust to repeated noise exposure, reducing the attention directed toward the noise. Attentional filtering is also probable, with the prefrontal cortex potentially enhancing its role in selectively attending to task-relevant information. Stress response modulation might involve changes in cortisol release and other physiological reactions to maintain stability. The combined effect of these factors may have resulted in the non-significant impact of noise type on attention and mental workload in this study. The experimental results did not support hypothesis H2e; instead, steady noise was found to induce mental fatigue more readily than complex noise. Continuous noise triggers a sustained stress response. Prolonged exposure to continuous noise can lead to chronic stress responses, resulting in the release of stress hormones such as cortisol and adrenaline. While these hormones for managing immediate threats, their long-term release can disrupt proper energy allocation, ultimately increasing feelings of fatigue (Heidari et al., 2021; Mucci et al., 2021). In contrast, although intermittent noise also elicits a stress response, it offers brief recovery periods on account of its irregular nature. These recovery periods can mitigate the cumulative effect of prolonged stress responses and alleviate overall fatigue. As a result, steady noise is more likely to cause mental fatigue than complex noise.

### 6.2 The impact of noise levels on learning efficiency and cognitive state

In examining the impact of noise levels on learning efficiency, the experimental results did not support hypotheses H3a and H3b, indicating that variations in noise levels within the same type did not significantly affect behavioral indicators. This could be due to the gradual adaptation of construction workers to continuous noise, which may diminish its negative effects on learning. Previous studies have shown that individuals develop an adaptive response to persistent background noise, reducing its interference with cognitive tasks over time (Park et al., 2018). Moreover, construction workers might possess an enhanced tolerance to noise, enabling them to maintain a relatively high learning efficiency even within noisy environments. This noise tolerance is associated with factors including individuals’ cognitive resources, psychological state, and past experience (Lewkowski et al., 2018). For example, Chere and Kirkham (2021) demonstrated that household noise significantly impair adolescents’ executive functions, but this effect only becomes noticeable when the noise reaches a certain level. This suggests that the impact of noise on learning efficiency may also follow a threshold model, noise fluctuations within a certain range may not significantly impact learning efficiency, but once the noise level exceeds a specific limit, the impact becomes pronounced.

Regarding the impact of noise levels on cognitive state, the experimental results supported hypotheses H4c, H4e, and H4f. High levels of steady noise were shown to significantly reduce attention, suggesting that individuals find it difficult to maintain high levels of attention in a continuous high-level noise environment. There was a trend of increased mental stress in high levels of steady noise conditions, suggesting that prolonged noise exposure increases psychological strain. Additionally, mental fatigue was notably elevated in high-level complex noise environments. These results consistently reflect the detrimental effects of high-level noise on cognitive state, highlighting the importance of noise levels and types on workers’ psychological and cognitive well-being in the workplace. However, the results did not support hypothesis H4d, as high levels of noise did not significantly elevate mental workload. This finding is in line with Jafari et al. (2019) suggest that noise has a high threshold for influencing mental workload. Specifically, even at a noise level of 80 dB(A), mental workload did not significantly increase, possibly because the effect of noise on mental workload only becomes evident beyond a certain intensity. It is also possible that workers had adapted to lower levels of noise, with higher noise levels or extended exposure being required to substantially affect their mental workload. Further analysis suggests that individual noise tolerance and adaptability could moderate the impact of noise on mental workload (Smith, 1991).

To verify the validity of the EEG-derived metrics, a comparison was conducted between these metrics and the behavioral data. The existence of significant correlations between attention and reaction time, along with those between mental workload and reaction time, indicates that the EEG metrics are effective in mirroring the cognitive states associated with task performance. Moreover, these metrics can be differentiated from potential artifacts originating from the noisy environment.

## 7 Conclusion

This study explored the impact of noise on construction workers’ learning efficiency and cognitive states using EEG technology further examined the correlation between cognitive states and learning efficiency. The research disclosed how noise affects workers’ cognitive processes and learning performance. Moreover, the findings shed light on how noise disrupts workers’ cognitive functions and influences their ability to acquire and apply knowledge, while also affording a deeper understanding of strategies for enhancing learning efficiency and mitigating occupational health risks. This study not only presents new physiological evidence on the detrimental effects of industrial noise in construction settings but also provides scientific foundation for optimizing the work environment and promoting worker well-being. By linking cognitive states to learning outcomes, this study offers actionable insights into reducing noise-induced cognitive impairments and fostering healthier, more productive work conditions. The key research conclusions are outlined as follows:

1. At the same noise level, complex noise had a more significant negative impact on construction workers’ learning efficiency compared to steady noise. This finding highlights the importance of noise complexity in the workplace and its effect on learning efficiency, indicating that the characteristics of noise have a considerable influence on workers’ cognitive and behavioral performance. This provides scientific evidence for the development of more effective noise management policies and training programs, which can better protect workers’ physical and mental health and promote the healthy development of the construction industry.
2. The impact of noise type on certain cognitive state indicators was significant. Specifically, steady noise was more likely to induce mental fatigue in workers, while complex noise was more likely to increase mental stress. This suggests that in a work environment, it is important not only to consider the intensity of noise but also to pay attention to the type and characteristics of noise in order to develop more effective intervention measures and management strategies. These strategies can help reduce the adverse effects of noise on workers’ psychological and cognitive health, thereby enhancing overall work efficiency and worker well-being.
3. High levels of noise can lead to deteriorated cognitive states, particularly in terms of decreased attention, increased mental fatigue, and heightened mental workload. Therefore, when developing noise management strategies, it is important to consider the long-term effects of noise intensity on workers’ cognitive functions.
4. Attention, mental workload, and mental stress were found to have a significant correlation with reaction time. This indicates that cognitive state acts as a crucial link between noise interference and learning efficiency, with different cognitive states influencing workers’ responses and performance in noisy environments. Consequently, management strategies aimed at mitigating the impact of noise on learning efficiency should include monitoring and regulating cognitive states to alleviate the negative effects of noise on workers’ learning and work, ultimately improving overall learning outcomes and work efficiency.

Owing to the restrictions imposed by the Ethics Committee regarding noise levels in human experiments, and taking into account the actual noise levels prevalent in the component factory, the present study solely investigated the impact of three levels of construction industrial noise, specifically 60 dB(A), 70 dB(A), and 80 dB(A), on construction workers’ learning efficiency and cognitive state. Noise levels surpassing 80 dB(A) were excluded from the study, as they might potentially inflict uncontrollable harm upon the human body. Future research will focus on leveraging state-of-the-art simulation tools to mimic the acoustic conditions of more extreme noise levels in construction settings. This will enable us to better understand the complete range of effects and develop more refined noise management approaches. Future research endeavors should strive to augment the sample size and make more meticulous distinctions among different noise types and sources. By doing so, it would be possible to conduct a more in-depth exploration of the specific effects and underlying mechanisms through which diverse types of noise influence learning efficiency and cognitive performance.

## Data Availability

The raw data supporting the conclusions of this article will be made available by the authors, without undue reservation.
